# Health system weaknesses constrain access to PMTCT and maternal HIV services in South Africa: a qualitative enquiry

**DOI:** 10.1186/1742-6405-8-10

**Published:** 2011-03-03

**Authors:** Courtenay Sprague, Matthew F Chersich, Vivian Black

**Affiliations:** 1 Graduate School of Business Administration, University of the Witwatersrand, Johannesburg, South Africa; 2 Centre for Health Policy, School of Public Health, University of the Witwatersrand, Johannesburg, South Africa; 3 International Centre for Reproductive Health, Department of Obstetrics, Ghent University, Belgium; 4 Wits Institute for Sexual and Reproductive Health, HIV and Related Diseases, Dept of Obstetrics and Gyaenocology, University of the Witwatersrand, Johannesburg, South Africa

## Abstract

**Background:**

HIV remains responsible for an estimated 40% of mortality in South African pregnant women and their children. To address these avoidable deaths, eligibility criteria for antiretroviral therapy (ART) in pregnant women were revised in 2010 to enhance ART coverage. With greater availability of HIV services in public health settings and increasing government attention to poor maternal-child health outcomes, this study used the patient's journey through the continuum of maternal and child care as a framework to track and document women's experiences of accessing ART and prevention of mother-to-child HIV transmission (PMTCT) programmes in the Eastern Cape (three peri-urban facilities) and Gauteng provinces (one academic hospital).

**Results:**

In-depth interviews identified considerable weaknesses within operational HIV service delivery. These manifested as missed opportunities for HIV testing in antenatal care due to shortages of test kits; insufficient staff assigned to HIV services; late payment of lay counsellors, with consequent absenteeism; and delayed transcription of CD4 cell count results into patient files (required for ART initiation). By contrast, individual factors undermining access encompassed psychosocial concerns, such as fear of a positive test result or a partner's reaction; and stigma. Data and information systems for monitoring in the three peri-urban facilities were markedly inadequate.

**Conclusions:**

A single system- or individual-level delay reduced the likelihood of women accessing ART or PMTCT interventions. These delays, when concurrent, often signalled wholesale denial of prevention and treatment. There is great scope for health systems' reforms to address constraints and weaknesses within PMTCT and ART services in South Africa. Recommendations from this study include: ensuring autonomy over resources at lower levels; linking performance management to facility-wide human resources interventions; developing accountability systems; improving HIV services in labour wards; ensuring quality HIV and infant feeding counselling; and improved monitoring for performance management using robust systems for data collection and utilisation.

## Background

In 2002, a national programme to prevent mother-to-child transmission of HIV (PMTCT) was established in South Africa, followed by an antiretroviral treatment (ART) initiative in 2004. To enhance ART access for pregnant women and address high mortality among women and children, eligibility criteria for ART initiation were revised in April 2010 to include all women with a CD4 cell count below 350 cells/mm^3 ^[1,2]. This marked a notable departure from previous ART criteria of an AIDS-defining condition or a CD4 count below 200 cells/mm^3 ^[3,4], and is consistent with WHO guidelines and evidence of survival benefits with earlier ART initiation [5-7].

Despite these prevention and treatment initiatives, HIV remains responsible for roughly 40% of mortality in South African pregnant women and children [8]. Within functioning health systems, PMTCT interventions can virtually eliminate HIV infection in infants. Countries such as Brazil, Botswana, the United Kingdom and United States have reduced rates of vertical transmission to below 2% [9-11]. Yet South Africa has achieved little success, holding the dubious distinction of having the greatest burden of HIV-infected children of any country [12]. If current trends persist, health and development targets will remain unattainable - including millennium development goals 4, 5 and 6 [13].

Within a context where HIV services are available in public facilities and government's attention to maternal-child health is increasing, we investigated the barriers facing pregnant women seeking access to these services. Using qualitative methods, we sought the perspectives of both patients and providers to illuminate aspects of the journey women take through the continuum of care, from pregnancy through to child health services.

## Methods

### Study sites and selection

The choice of study sites was purposive, aiming to compare different settings, including peri-urban, resource-limited areas of the Eastern Cape Province and an urban setting in Gauteng Province. Though the provinces have a similar HIV prevalence (30% among pregnant women), they have marked differences. In 2008, 70% of the 6.4 million residents of the Eastern Cape were classified as poor, 30% as unemployed and 94% received care in the public health system [14]. Gauteng's population is larger (an estimated 10.5 million), with better socio-economic indicators: fewer are classified as poor (42%), unemployed (23%), or reliant on public health services (78%) [15].

The study took place between March 2008 and February 2009. Four public sector facilities were studied, namely: an academic hospital in Johannesburg, Gauteng; and in the Eastern Cape, an academic hospital, a regional hospital and a primary health care clinic. The Eastern Cape facilities only began implementing ART for pregnant women midway through the study, as recommended in 2008 national guidelines; whereas the Johannesburg facility had already done so in early 2008 [16].

Ethics approval was granted by both provincial departments of health, by the Human Research Medical Ethics Committee of the University of the Witwatersrand (protocol number M080119) and Walter Sisulu University, Eastern Cape (protocol number 00032-07). All interviewees gave informed consent. Where individuals gave consent for recording, interviews were audio taped. About 40 respondents, across respondent categories, declined to be taped, likely due to concerns about confidentiality of their views, with health personnel perhaps fearing how the taped information might be used and possible punitive action in their workplace.

### Data collection and analysis

To allow for triangulation, in-depth interviews were undertaken with patients (83 HIV-positive women); caregivers (32 female caregivers of HIV-positive children); and key informants (38), including HIV and public health specialists, academics, nurses, doctors and HIV lay counsellors.

Patients' files (n = 83) were reviewed, allowing for an independent assessment of health provider action and HIV services delivered during antenatal care, childbirth and postpartum. Where available, socio-demographic data (e.g., income, access to electricity, piped water and flush toilet) and HIV management (ART regimen, counselling notes and PCR testing of infants) information were extracted.

All interviews were done by the principal investigator with translators present during interviews - which if in isiXhosa or isiZulu - were translated immediately into English to allow for probing. Interview transcripts and patient data were reviewed by the investigators and, using grounded theory, key themes and core categories were documented as they emerged, aiming to reach data saturation [17].

### Qualitative approach

The rationale for selecting qualitative methods is that previous research in South Africa has predominately focused on quantitative measures of PMTCT 'coverage'. This has included examining barriers to rolling out a minimum package of services for pregnant women. Several authors have documented PMTCT performance against numerical targets, mainly within the 'PMTCT cascade', and broadly assessed programme effectiveness [18,19]. While undoubtedly important, existing research has neglected the often fraught interface between patients and the health system - particularly women's experience of health services and her consequent health-related behaviour (e.g., returning for repeat ANC visits or dropping out of the public health system). Such behaviour is undeniably rooted within larger contexts of socio-cultural norms (e.g., around breast feeding and HIV stigma) as well as the harsh economic realities facing women with HIV. This nexus between individuals and systems fundamentally impacts on the degree to which a pregnant woman is able to benefit from prevention and treatment interventions. Against that background, qualitative methods were employed to understand women's experiences of HIV services, and of delays or impediments to these services.

## Results and Discussion

In-depth interviews identified considerable weaknesses within operational systems for delivering PMTCT and ART in all four facilities. In tracking a woman's journey from antenatal care (ANC) through to paediatric HIV care, the study documented a series of delays, coupled with a lack of access to information and support at key points in the care continuum. Several broad themes emerged in analysis. These are grouped in the sequence of care and followed by a number of cross-cutting issues. Pertinent background information is added where necessary to set the context.

### The Care Continuum

#### Antenatal Care

##### Shortages in staff and supplies delay HIV testing for pregnant women

HIV testing within ANC is the entry point to the care continuum for pregnant women. Across the facilities studied, a significant proportion of the HIV-positive pregnant or postnatal women interviewed failed to receive an HIV test during their first ANC visit, mainly due to shortages in staff and supplies. In both Eastern Cape hospitals, nurses provided all counselling and related HIV services, with a single nurse per facility running the PMTCT programme and offering all HIV counselling. In addition to their other duties, the 'assigned' nurses provided PMTCT services for about five hours a day (8:30 am to 1:30 pm) from Monday to Friday. As a means of coping with this workload, one nurse explained: "I provide five counselling sessions per day, and then I stop [because] I have other work to do" (Eastern Cape hospital, October 2008). If this nurse was ill or undergoing training elsewhere, HIV services were simply not available. Infrequently, an HIV counsellor or doctor would assist in providing some counselling, although many respondents believed that doctors were too busy to provide optimal counselling. The nurses acknowledged that there was generally, then, no HIV testing and counselling provided for patients admitted during the afternoons, weekends, or on public holidays.

Such nursing shortages are evident throughout the country. In 2008, for example, Health Systems Trust data documented a nurse staffing gap of 36% for public sector posts nationwide and 40% for the Eastern Cape, with some provincial deficits registering upwards of 50% [20]. Nurses interviewed spoke of the challenge of attracting and retaining health personnel in the Eastern Cape, especially in certain peri-rural towns. At the Eastern Cape tertiary-facility, only half of the 600 nursing posts were filled. Respondents there stated that it was commonplace for nurses to depart for more promising posts in the private sector or overseas. In the month when interviews took place, three nurses at the facility were leaving at that month-end alone (interviews with key informants, Eastern Cape academic hospital, October 2008).

In the Eastern Cape clinic, shortages of HIV test kits and stock-outs of nevirapine, were reported by staff. The popular press, together with academic sources, found similar problems with drug procurement and supply bottlenecks in other parts of the country [21-23] (see Table 1, Table 2). By contrast, according to both patients and key informants, the hospitals in the Eastern Cape and Johannesburg had no such supply problems. However, in Johannesburg, systems' failures took the form of frequent delays in payment to lay HIV counsellors who were responsible for testing and counselling. Absenteeism and low staff morale were common. Indeed, over the past few years a leading South African NGO, the AIDS Law Project (now operating under the name SECTION27), had called for the Department of Health to address the poor employment conditions of lay counsellors, pressing for legal action to address this chronic problem [24-26].

**Table 1:** Avoidable health personnel and systems barriers to ART and PMTCT in four facilities in South Africa: the maternal-child care continuum

***Antenatal Care***	
**HEALTH PERSONNEL**	
**HIV Testing & Counselling**	**In Which Facilities?**
HIV counsellor unavailable for testing	All four
Repeat testing unavailable for patients who had earlier declined	All four
Counsellor unavailable at time of HIV test	All four
**ART Eligibility/Initiation**	**In Which Facilities?**
Health staff miss ART eligibility in patient's file	All four
**HEALTH SYSTEM**	
**HIV Testing & Counselling**	**In Which Facilities?**
No HIV test kit available	Eastern Cape clinic only
**ART Eligibility/Initiation**	**In Which Facilities?**
Patient file does not have CD4 cell result	All four
***Labour Ward***	
**HEALTH PERSONNEL**	
**HIV Testing & Counselling**	**In which Facilities?**
No counselling for HIV positive woman on infant testing at six weeks; ART for woman and infant; immunization; cotrimoxazole; nutrition; family planning; safer sex; partner testing	All three hospitals
**HIV Prevention**	**In which Facilities?**
No ARV prophylaxis given to HIV positive woman in labour	All three hospitals
Infant not given ARV prophylaxis when mother's HIV positive status is clear	Johannesburg hospital
**HEALTH SYSTEM**	**In which Facilities?**
**HIV Testing & Counselling**	
Woman with unknown HIV status not tested	All three hospitals
Woman's HIV status unclear from file	All three hospitals
***Postnatal Care (after patient is discharged and returns for follow up care)***	**In which Facilities?**
**HEALTH PERSONNEL**	
**HIV Testing & Counselling**	
Woman with unknown HIV status not tested	All three hospitals
HIV positive woman fails to take child for PCR test	Johannesburg and Eastern Cape academic hospitals (only these facilities offer PCR testing)
**HEALTH SYSTEM**	
**ART Eligibility/Initiation**	**In which Facilities?**
Woman with HIV does not receive CD4 cell test	All three hospitals
***Paediatric Ward (only pertains to Eastern Cape Academic Hospital and Johannesburg Hospital)***	
**HEALTH PERSONNEL**	**In which Facilities?**
**HIV Testing & Counselling**	
HIV-exposed child admitted for TB not tested for HIV	Johannesburg hospital
HIV positive child's mother with status unknown not referred for HIV testing	Johannesburg and Eastern Cape academic hospitals
**ART Eligibility/Initiation**	**In which Facilities?**
ART eligibility of mother (with HIV positive child unknown)	Johannesburg and Eastern Cape Academic hospitals

**Table 2:** Women's perspectives on barriers to ART and PMTCT: reported barriers which delayed or denied HIV prevention and treatment

*Individual barriers*	*Reported in the following sites*
No money for transport	Johannesburg hospital, Eastern Cape clinic
Fear of positive HIV test	All facilities
Denial of positive HIV result (i.e., received positive result but did not trust the result	Johannesburg hospital, Eastern Cape academic hospital
Refused testing	All facilities
*Health personnel*	
Judgmental attitude	All facilities
Stigmatizing attitude (name calling, blame, shunning)	Johannesburg hospital, both E. Cape hospitals
No health personnel available to provide HIV testing	All facilities
No health personnel available to provide counselling (e.g., regarding treatment and infant feeding options)	All facilities
Clerk turns patient away at first booking	Johannesburg hospital
Health personnel did not provide ARV prophylaxis during labour/delivery	All hospitals (clinic does not perform deliveries)
*Health system*	
HIV test kit not available	Eastern Cape clinic
Nevirapine stockout	Eastern Cape clinic

##### Delays in obtaining CD4 cell count results hinders ART initiation

Another consistent delay for HIV-positive women concerned the timely receipt of their CD4 cell count results, a necessary step for discerning ART eligibility. When women attended their second or third ANC visit, they often could not commence ART as their CD4 cell counts were still unavailable. Patient files indicated that many HIV-infected women, though eligible for ART, had already delivered before initiating ART or PMTCT prophylaxis, either due to the above-mentioned systems' failures, or, in some instances, preterm delivery. A further group of women began ART late - just prior to childbirth - making optimal prevention and treatment outcomes less likely [27].

#### Postnatal Care

##### Lack of healthcare worker knowledge impacts on safe infant feeding

Postnatal care constitutes the next component of the care continuum, where there are a number of opportunities for protecting the health of the woman and her newborn by optimizing HIV prevention and treatment. During breastfeeding, for example, the efficacy of ARV drugs taken in pregnancy and during labour is reduced over time, [28,29], with postnatal HIV transmission responsible for up to half of HIV infections in South African children. Mixed feeding carries a particularly high risk [30,31]. Feeding options need to be clearly explained and women counselled on the implications of their feeding choices during the early postnatal period. This study found that one of the weakest aspects of PMTCT interventions is counselling women on infant feeding. Across the facilities, many HIV-positive women struggled with feeding choices, with a number practicing mixed feeding, unaware of the increased risks of transmission. This reflects the poor and *ad hoc *counselling received by women during ANC and postnatally.

Based on interviews with pregnant or postnatal women, during 'counselling' about infant feeding options, healthcare workers in many instances appeared to 'steer' women towards their own preference, encouraging women to do what the health personnel believed to be 'right' or 'proper'. This often resulted in inappropriate choices given women's available resources - in terms of money, time, and access to safe water. For example, in one of the Eastern Cape hospitals, records showed that 97% of women in August 2008 and even 100% of women in September of that year elected to formula feed. While free formula is available in clinics across the country, only 9% of households in the surrounding district have potable water [32] - meaning that women in this district would struggle to ensure safe formula feeding. One woman observed: "I wasn't given feeding options - I was simply told to formula feed" (Johannesburg, May 2008). Another said: "The nurse told me that formula feed was the only safe option - she did not give me a choice" (Johannesburg, June 2008).

#### Infant Diagnosis And Care

##### Ensuring early HIV diagnosis remains challenging

Infant HIV diagnosis is critical, especially early diagnosis (and subsequently ART if required), but has proved challenging in South Africa [33]. Organisation of services in a vertical manner accounts for much of these difficulties, together with the related problem of limited locations for testing infants in the these peri-urban facilities.

In the Johannesburg site, polymerase chain reaction (PCR) testing required mothers to take their infants to the paediatric virology ward, a different location from where they had attended antenatal and postnatal care but within the same facility. In the Eastern Cape, women were required to attend an entirely different hospital, as only the academic hospital in the district offered PCR testing. In both settings, health personnel were meant to direct women accordingly, however, many women appeared unaware of this information.

#### Crosscutting Issues Throughout The Care Continuum

##### Stigma

A former nurse interviewed in the Eastern Cape clinic noted that the 'tins' used for formula feeding were associated with stigma (October 2008). This was confirmed by patients and health personnel interviewed, and has been identified in previous studies [34]. One woman noted: "I hide it [her HIV-positive status]. I say the baby doesn't like breast milk to anyone who asks why I am not breastfeeding" (Johannesburg, August 2008). On this theme, another woman, when asked how she managed formula feeding, said: "I put the formula in a canister without a label (e.g., a can for instant coffee). I worry about what people think, so I cannot tell anyone about my status outside my family. I keep it to myself" (Johannesburg, August 2008).

One health worker also noted: "People are scared of themselves" and "stigma prevents people from testing" (Johannesburg, August 2008). Attributing her experiences to discrimination, one pregnant woman stated: "I was turned away at X and Y clinics. I was already on ARVs. Maybe they turned me away because I was HIV positive?" (Johannesburg, June 2008).

While patients, healthcare workers and researchers agree that stigma is abating somewhat, it remains pervasive. *Human Rights Watch *noted: "People living with HIV and AIDS in South Africa continue to fear discrimination and victimisation. Few people choose to publicly disclose an HIV-positive status, fearing that this will cause stigmatisation in their community and loss of their jobs" [35].

##### Health personnel-patient interaction and psycho-social support

Women's HIV status has an impact on their mental health, which can then affect their willingness and ability to seek health services and care [36]. Shock, denial or uncertainty can delay women's return to health facilities for the next step in HIV service provision, namely, ART initiation. While such individual barriers may be difficult to obviate completely, comprehensive counselling can mitigate this. One woman stated: "There is stigma attached to HIV. I cope by not telling people because people will criticize. I gain support from one of the counsellors at the hospital when I feel low" (postnatal patient, Johannesburg, June 2008). An antenatal patient said: "Being HIV positive was difficult at first. But since I have had HIV counselling here I feel strong because of the counselling" (Johannesburg, April 2008). Patients described how, when they did see a nurse or counsellor, health personnel would share strategies about adherence, disclosure and how to deal with in-laws who disapprove of women who do not breastfeed. At other times, however, health personnel played a more directive, even invasive, role. One patient reflected: "I was in denial about going onto ARVs and refused at first. It was only when I went back to a second HIV counsellor that she said 'you are killing your child by not taking the medication'". The patient then "became very worried about the baby's health and I was frightened into action". After she told her husband she was taking ART: "He reacted in a violent manner and threw the pills away". The counsellor then helped the woman put "the pills in a different place to take the pills in secret" (Johannesburg, July 2008).

Thus, though there were many examples of health workers seemingly being overwhelmed by their workload and working conditions, this did not always prevent them from offering assistance to women, often drawing on years of experience from previous interactions with HIV-infected women. Health personnel who knew the patient's status - and offered guidance about the complex challenges facing HIV-positive women in South Africa - were thus able to play a critical support role for some women in this study.

##### Inadequacy of data and information systems for monitoring and evaluation

The facility in Johannesburg kept routine statistics, which were computerised. However, in the Eastern Cape, there were no computers at two of the three facilities, and information was recorded manually. The type of indicators recorded - and the actual figures tallied - seemed to reflect only a portion of the actual PMTCT and ART activity, and the data were generally of poor quality. Consequently and likely perpetuating these poor monitoring practices, what little information health staff collected was not being used to improve current practices and systems: "There are no feedback loops for quality improvement" as one key informant noted (Eastern Cape hospital, June 2009).

**Figure 1:**
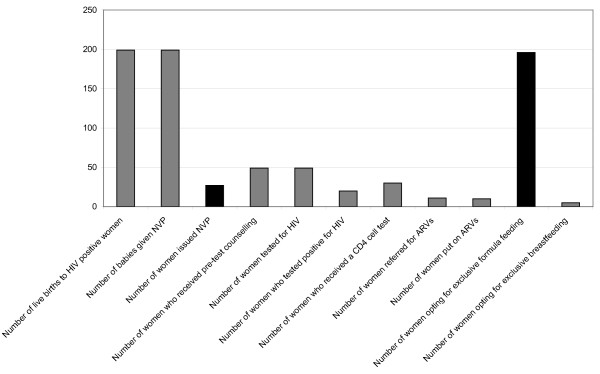
**PMTCT Indicators Recorded for August and September 2008 at an Eastern Cape Facility**. • DHIS indicator performing extremely poorly. • DHIS indicator

In the Eastern Cape (non-academic) hospital, in terms of HIV testing, the numbers of women who apparently tested for HIV were 24 in August and 25 in September (Figure 1). Yet there were a total of 107 live births to HIV-positive women who delivered in August; and 92 in September. Over the two months, of the 24 women tested, 11 women (or 50%) tested positive for HIV in August; and in September, of the 25 women tested for HIV, 9 women (26%) were HIV positive: but only 13 women (12%) in August and 14 (15%) in September were apparently issued NVP. In the figures available, only 2% of women in August (n = 107) and 9% of women in September (n = 92) were initiated onto ART. The figures, however, seldom tally. For example, in August, the number of women choosing to exclusively breastfeed plus formula feed equals 109 while the total number of HIV-positive women giving birth during August is 107 (two more than the total). This suggests double counting, incorrect counting and generally poor record keeping.

Overall, in terms of data availability and quality, one data capturer said that 70% of the data were simply not recorded at all (Eastern Cape Province, October 2008). Referring to this province, another key informant observed: "Data quality is very poor across the province". "Statistics in the nevirapine register are accurate" but "some statistics are double-counted" and "they carry over figures from the previous month". Health personnel fill in information, but they are not working from a common definition of an indicator (Eastern Cape hospital, October 2008). Ultimately, the actual performance of the PMTCT and ART programme in the Eastern Cape facilities appears largely unknown.

## Conclusions

The study found many instances where opportunities for HIV testing were missed in antenatal care, diminishing any chance of a care continuum. Most obvious missed opportunities stemmed from shortages of staff and test kits. Further, opportunities for preventing HIV are not maximised in labour wards, and counselling to reduce postnatal transmission during infant feeding is generally inadequate. Moreover, paediatric HIV testing, the gateway for infant testing and care, remains under-utilised. Even in the Johannesburg facility, the most-resourced hospital, a series of systems and individual factors delayed HIV services for pregnant women. These factors are interdependent: a single delay reduces the likelihood of women accessing ART and PMTCT, but delays occurring in tandem often signal a comprehensive denial of prevention and treatment.

Health personnel comprise the critical link between patients and health systems. Our analysis suggests that there is great scope for health systems' changes, much of which centres on health personnel capacity and performance. To better address the needs of HIV-positive pregnant or postnatal women, site-specific recommendations include: reviewing HIV staffing levels in the Eastern Cape and ensuring a sufficient number of conventional or lay staff is assigned to HIV service provision. In that province, human resource policies, planning and training must focus on recruitment and retention, attending to shortages of personnel in rural and peri-urban areas, while other interventions at the facility-level should address working conditions, offer incentives and provide professional development opportunities. Evidence on improving productivity, competence and responsiveness of health workers indicates that specific elements should be included, such as ensuring autonomy over resources at lower levels; linking performance management interventions to facility-wide human resources management; and developing accountability systems to ensure that health workers and managers are responsible for their performance [37].

In the Johannesburg site, lay counsellors must be assured proper payment and conditions of service, including regular pay, debriefing, training and career pathing. Further, improved communication and referral networks are required between antenatal care, postnatal care and paediatric units in the same facility.

This study shows that women often look to health providers for information, answers, comfort, counselling and support - not only for physical ailments but for psychological distress related to their HIV status, including stigma. Mental health is much-neglected in South Africa generally, and particularly for women [38]. South African women face conditions of poverty, gender inequality and social disadvantage. In addition to living with HIV, women may suffer from intimate partner violence or other forms of abuse. Nurses and social workers, in particular, can assist women to navigate the myriad challenges they face and address their mental health, including maternal and postnatal depression and other anxiety and stress-related disorders. On-site support groups and health worker advice with coping are important sources of psycho-social assistance.

Across the four facilities, the training and repeat training of health personnel (nurses and lay counsellors) in quality HIV and infant feeding counselling is essential. Improved monitoring and evaluation for performance management are equally important in enhancing service delivery [39]. In South Africa, van der Merwe et al. underscore that strengthening linkages and integrating key components of ART within antenatal care reduces "time-to-treatment initiation" for pregnant women [40]. Others have advocated for strengthening of facility supervision with emphasis on the use of antenatal and labour-ward checklists to record and monitor facility activities. They also emphasize the role of data collection, analysis and utilization to improve health services [41]. Equally, Chopra et al. recommend building "a culture of using data to improve care" in South Africa [42].

The study has several limitations. These include potentially incurring reporting bias, as interviews within clinical sites might cause patients to downplay negative experiences due to fear of poor subsequent treatment from the hospital, even though consent forms explicitly emphasized confidentiality. Further, the analyses, interpretation and conclusions may not be generalisable to other parts of the country, even though many findings were common across the two sites.

Finally, to achieve improved maternal, newborn and child health, it is critical to exploit the opportunities for preventing HIV in children and treating HIV in women and children at all points in the care continuum [43-48]. Using evidence-based approaches to address the identified gaps in the health system is a necessary first step in ensuring that women and children benefit from HIV services that are presently available, yet remain out of reach for too many South African women and children.

## Competing interests

The authors declare that they have no competing interests.

## Authors' contributions

CS carried out the interviews, conceived the study and drafted the first manuscript. VB participated in study conception, design, execution, coordination and helped to draft the manuscript. MFC assisted in drafting the manuscript and gave critical review. All authors read and approved the final manuscript.
